# Case report: A rare low-grade fumarate hydratase-deficient renal cell carcinoma

**DOI:** 10.3389/fonc.2024.1440119

**Published:** 2024-11-26

**Authors:** Di Sun, Baohong Hu, Xinna Li, Ping Yang, Guohua Yu

**Affiliations:** ^1^ Department of Pathology, Yantai Yuhuangding Hospital of Qingdao University, Yantai, China; ^2^ Department of Oncology, Yantai Yuhuangding Hospital of Qingdao University, Yantai, China

**Keywords:** renal cell carcinoma, FH-deficient, low grade, case report, literature review

## Abstract

**Aim:**

To study and analyze the clinicopathological features of low-grade fumarate hydratase-deficient renal cell carcinoma in order to improve the understanding of the diagnosis and management of rare and specific morphological cases of this tumor.

**Methods:**

A case of low-grade FH-deficient renal cell carcinoma in a male is reported, and its clinicopathological features were analyzed and literature review was performed.

**Results:**

A 48-year-old middle-aged male with no relevant positive clinical signs was admitted to the hospital with an incidental finding of a mass in the lower pole of the left kidney during abdominal CT examination. Pathomorphology showed that the tumor cells were arranged in a variety of structures and the cells were low grade eosinophilic. Immunohistochemistry showed Pax-8 (+), Vim (partially +), FH (-), CK7 (-), CD117 (-), P504s (partially +), CD10 (partially +), CA-9 (-), TFE3 (partially +), SDHB (+), CK20 (-), and Ki-67 (+, about 2%). Final pathologic diagnosis: FH-deficient renal cell carcinoma (low grade).

**Conclusion:**

Single pure low-grade FH-deficient renal cell carcinoma of the kidney is extremely rare, and the image structure of this tumor exhibits diverse manifestations, which needs to be differentiated from many renal tumors in clinicopathological diagnosis in order to prevent misdiagnosis.

## 1 Introduction

Fumarate hydratase-deficient renal cell carcinoma (FH-deficient RCC) is a rare type of renal cell carcinoma (RCC) caused by alterations in the fumarate hydratase (FH) gene. Germline mutations in the FH gene can cause autosomal dominant tumor syndromes with characteristic lesions manifesting as skin and uterine leiomyomatosis and renal cell carcinoma (1). Due to the unique morphological and molecular genetic features of this tumor, the 2016 edition of the WHO renal tumor classification included it as an additional renal cell carcinoma subtype and named it hereditary leiomyomatosis and renal cell carcinoma (HLRCC) (2). In the 2022 edition of WHO, it was pointed out that FH somatic mutations may also lead to the development of this type of renal cell carcinoma, and have extremely similar biological behaviors to HLRCC caused by FH germline mutations. Therefore, renal cell carcinomas caused by FH germline or somatic mutations have been collectively referred to as FH-deficient renal cell carcinomas in the new 2022 edition of the WHO renal tumor classification (3).

FH-deficient renal cell carcinoma is pathologically arranged in a variety of structures, and the classical histological images show a high-level morphology: cells with abundant cytoplasm and large nuclei, with prominent viral inclusion body-like eosinophilic macronuclei and perinuclear empty halos seen in some cases (2), which is suggestive of the diagnosis of this tumor. Previously, FH-deficient renal cell carcinoma was usually recognized as a highly aggressive tumor, prone to local progression and early metastasis, with a poor prognosis (4). With further research, the 2022 edition of WHO proposed that a few cases of FH-deficient renal cell carcinoma are eosinophilic low-grade forms, but the description is relatively brief, and most of these rare form of tumors have a relatively favorable prognosis (3). The ever-expanding histologic picture of FH-deficient renal cell carcinoma has further increased the difficulty of diagnosing this disease, and in practice, it needs to be differentiated from more types of renal tumors. We report a case of low-grade FH-deficient renal cell carcinoma in a middle-aged male and conduct a literature review to improve the understanding of the diagnosis and management of rare cases of this tumor in pure low-grade form.

## 2 Case description

### 2.1 Patient details and initial diagnosis

**Figure 1:**
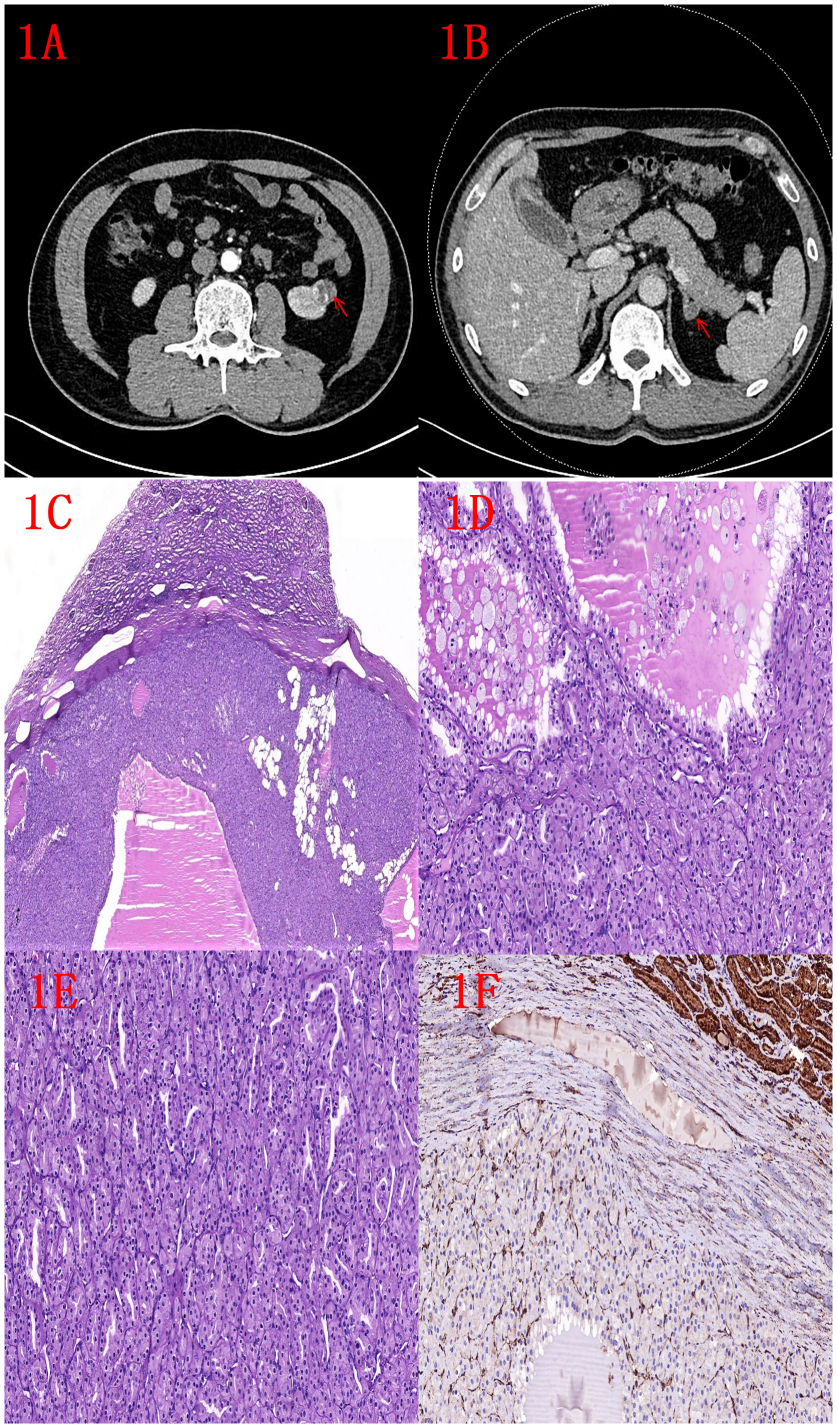
**(A)** CT image of left kidney tumor (indicated by red arrow). **(B)** Image of left adrenal tumor (indicated by red arrow). **(C)** HE image of the left kidney tumor: low magnification shows a well defined tumor with an outer pseudo-envelope and a focal fatty component (2x). **(D)** HE image of left kidney tumor: nested and microcystic structures, foam cells visible within microcystic structures (10x). **(E)** HE image of left kidney tumor: tubular and nested structures (10x). **(F)** FH immunohistochemistry: negative expression of renal tumor and positive expression of normal renal tubules (10x).

Informed consent has been obtained from the participant and the study was approved by Ethics Committee of Yantai Yuhuangding Hospital. The patient was a 48-year-old man who had been admitted to a local hospital three months earlier for cerebral infarction, and a mass in the lower pole of the left kidney was detected during a routine abdominal CT examination. The patient had no obvious pain in the lower back and abdomen, no hematuria in the naked eye, no uncomfortable symptoms such as urinary frequency, urgency, and pain of urination, and no obvious abnormality in the skin and mucous membranes of the whole body. The patient was admitted to our hospital for further treatment, and CT examination of the abdomen in our hospital showed that rounded mass was seen in the lower pole of the left kidney, with a size of about 2.1 cm × 1.6 cm, and the lesion protruded outside the renal silhouette (degree of convexity ≥50%) (**Figure 1A**). In addition, rounded low-density mass shadow was seen in the left adrenal region, with a diameter of about 1.1 cm (**Figure 1B**). The patient had a history of hypertension for more than 20 years. There was no past history of surgery, no similar patients in the family, and the genetic history was denied. Laparoscopic partial nephrectomy (left side) + laparoscopic resection of adrenal lesion (left side) were performed after completing relevant clinical examinations.

### 2.2 Pathological features

Gross description:

Marked left kidney tumor: The submitted left kidney tissue measures 2.1 × 2 × 2 cm. The cross-section reveals a solid mass measuring 2.1 × 2 × 1.7 cm, with clear demarcation from surrounding tissues and approximately 0.3 cm from the renal capsule. Surrounding the mass are small amounts of adrenal and adipose tissue.

Marked left adrenal tumor: An irregularly shaped specimen measuring 5.5 × 3 × 2 cm. The cut surface displays a golden-yellow mass measuring 1.1 × 1 × 0.7 cm.

Microscopic features of the left renal tumor: the tumor had clear borders with a pseudo-envelope, the cytoplasm of the tumor cells was eosinophilic, arranged into nests, tubes and microcystic structures, foam cells could be seen in the microcystic structures of the foci, the cells were in the form of a completely low-grade morphology, and a small amount of adipose component could be seen in the foci (**Figures 1C-E**).

Pathological diagnosis: (Labeled left kidney tumor) FH-deficient renal cell carcinoma (low grade) without invasion of the renal peritoneum, no tumor involvement at the margins. Immunohistochemistry: Pax-8 (+), Vim (partially +), FH (-), CK7 (-), CD117 (-), P504s (partially +), CD10 (partially +), CA-9 (-), TFE3 (partially +), SDHB (+), CK20 (-), and Ki-67 (+, about 2%) (**Figure 1F**). (Labeled left adrenal tumor) Adrenocortical adenoma.

Molecular detection (Next-generation sequencing method):

**Table:** 

Gene	Mutation	Mutant	Abundance
FH	c.555 + 1G>C Intron 4 Splicing Mutation	c.555 + 1G>C	52.90%

Explanation: The splice-site mutation reported in this case was approximately 50% (heterozygous), suggesting that the tumor cell purity in the analyzed sample was around 50%. This may be due to the presence of stromal, immune, or other non-tumor cells within the sample, resulting in the FH mutation being detected at only 50%.

## 3 Follow-up and outcomes

As of August 2024, the patient is in good condition.

## 4 Discussion

Conventional fumarate hydratase-deficient renal cell carcinoma (FH-deficient RCC) is usually considered a high-grade, aggressive subtype of RCC that is frequently seen in the hereditary leiomyomatosis renal cell carcinoma (HLRCC) (5). Newer studies have found that purely somatic mutations in the FH gene may also lead to FH-deficient RCC, and that these patients without germline mutations will not exhibit the other symptomatic manifestations of HLRCC, and that specific FH mutations do not appear to alter the aggressive behavior and poor prognosis associated with this tumor (4). With increasing research experience, it has been recognized that the histological spectrum of FH-deficient renal cell carcinoma has expanded from high-grade to low-grade tumors, with occasional reports of synchronous and metachronous conventional high-grade morphology in low-grade forms (4, 6). The clinicopathological features of classical high-grade FH-deficient RCC are now well known, and the typical high-grade morphology region in the rare coexisting tumors of low-grade and high-grade morphology has a certain suggestive significance for the diagnosis of this tumor, and is similar to the poor prognosis of tumors with pure high-grade morphology (4, 6), which will not be repeated in this paper.

Based on the expansion of the 2016 WHO New Classification of Male Urologic Renal Tumors, a total of 15 cases of pure low-grade FH-deficient RCC were screened in retrospective studies and routine differential diagnosis by many scholars in recent years, and some of them were previously classified as other renal tumors, such as unclassified renal cell carcinoma (7) or oncocytoma (8), which were initially reported and described as resembling “SDH-deficient RCC morphology” (6) by Smith et al. The clinicopathological features of these 15 rare cases are summarized as follows (**Table 1**) (4–10): (1) there was no significant gender preference (female: male 8:7); (2) the age of onset was relatively young 10-66 years (mean age 27.6 years), with 5/15 cases younger than 20 years; (3) 1/15 men had symptoms of left back and abdominal pain, 2/15 women had symptoms of menstrual changes, pelvic and abdominal pain, difficulty with sexual intercourse, and fatigue, and 4/15 had incidental or syndromic screening findings on imaging; (4) no family history or evidence of HLRCC syndrome in 7/15 cases, 1/15 males with multiple cutaneous leiomyomas, and 4/15 females with personal and/or family history of uterine leiomyomas and renal tumors; (5) tumor size: 0.2-16.9 cm (including multilocularity in 2/15 cases); (6) the tumor were largely solid, cystic-solid and multilocular cystic/extensively cystic; (7) Pathologic images: a. Structural diversity: may appear solid, nested, tubular, tubulo-cystic, microcystic, cystic, occasionally fascicular, etc., usually with a mixed pattern of growth; b. Cellular morphology: 14/15 cases showed low-grade eosinophilic morphology (including 1 case with low-grade eosinophilic morphology in the first partial nephrectomy tumor, and high-grade morphology in the radically treated renal tumor 4 years later), and 1/15 cases showed low-grade spindle-shaped morphology; Except for one case of special spindle cell morphology, the cytoplasm of the tumor cells in the remaining 14 cases was abundantly eosinophilic with variable flocculation and vacuolization, and had monomorphic rounded nuclei with fine granular chromatin and inconspicuous nucleoli; The nuclei of some tumor cells may protrude into the lumen, giving a flathead nail-like appearance (9); c. concomitant phenomena, psammoma bodies were seen in 1/15 cases (8), fatty components were seen in 1/15 cases (in this case), and interstitial laxity edema was seen in 2/15 cases (5, 6); (8) Immunohistochemistry: all cases (15/15 cases) showed negative expression of FH; (9) Molecular testing: molecular testing was performed in 7/15 cases, and the results showed different FH gene alterations; (10) Other findings: 2/15 cases of renal tumors with concomitant adrenocortical adenomas; (11) Follow-up data: 12/15 cases with good prognosis, 3/15 cases with metastasis/death (including 1 case of high grade tumor metastasis with reoperation four years later, and 1 case of death from spindle-shaped tumor metastasis).

**Table 1:** Summary table of clinicopathological features of 15 cases of pure low-grade FH-deficient renal cell carcinoma.

Clinicopathological features											
	Year of publicatio n/author (number of cases)	Gender	Age	Surgical approach/size	First clinical symptoms	HLRCC evidence and family history	Histological images (complete low-grade morphology)	FH immunohistoch emistry	Molecular detection	Other Findings	Follow-up status
Case 1	2017/Smith et al(3 cases)	male	35	Surgical approach: radical nephrectomy Size: multiple,0.2- 9cm	Left back and abdominal pain	Multiple cutaneous leiomyomata	Structure : variable, solid,nested, tubular,microcysts Cells : cytoplasmic oncocytic (grade 2) cytoplasmic flocculence Interstitial mass : edema	Negative	NA	adrenocortica l adenoma, 2.7cm	NED, 34 months
Case 2		male	11	urgical approach: radical nephrectomy Size:8.5cm	Imaging for syndromal surveillance	Germline G354R FH mutation	Structural : Solid, tubular and cystic Cells : cytoplasmic vacuolation, hyaline inclusions (grade 2)	Negative	c.1189 G>A, p. Gly397Arg	NA	NED, >7 years
Case 3		male	41	Surgical pproach: partial nephrectomy,ra dical nephrectomy 4 years later Size: 5cm(radical)	Incidental findings by MRI	NA	Structure : Solid Cells : cytoplasmic vacuolation, rare hyaline inclusions ( grade 2)	Negative	NA	Multilocular cystic change ; later high grade FH-deficient RCC	NED, 4 years (oncocytic RCC) ; metastasis at 3 months after high grade RCC nephrectomy
Case 4	2018/Li,Y et al (4cases)	male	25	Surgical approach:NA Multifocal:4 nodules (0.4- 6.2cm)	NA	None	Structure : Nested, solid and cystic,oncocytoma-like Cells : Low-grade oncocytic Occasional psammoma bodies	Negative	NA	NA	NA
Case 5		female	10	Surgical approach:NA Size:5.5cm	NA	None	Structure : Nested tubular and cystic Cells : Low-grade Oncocytic	Negative	NA	Clinical history: Presented with hypertension	NED 24 months
Case 6		female	19	Surgical approach:NA Size:3cm	NA	None	Structure : Nested tubular ; Low-grade oncocytic	Negative	NA	NA	NA
Case 7		female	17	Surgical approach:NA Size: 16cm	NA	None	Structure : Nested,tubular Cells : Low-grade oncocytic		NA	NA	NA
Case 8	2019/Gupta male et al (2 cases)	male	22	Surgical approach:NA Size:3.8cm	NA	None	Structure : tubular Cells : low grade oncocytic features	Negative	NA	NA	Lymph node metastasis, died of the disease in 10 months
Case 9		male	66	Surgical approach:NA Size: 12.5cm	NA	None	Structure : fascicles Cells : low grade spindle cell	Negative	NA	NA	Lymph node metastasis, died of the disease in 29 months
Case 10	2019/Pan,X et al (2cases)	female	26	Surgical approach:NA Left size : 3.5cm Right size: 0.9cm	NA	Bilateral renal tumours ; uterine leiomyoma ; her mother accepted hysterectomy for multiple leiomyomas at 38 years	Left side structure : Nested,tubular,microcys ts Right side structure : solid Cells : cytoplasmic oncocytic (grade 2) Foam cells are seen in the left focal canalicular lumen	Negative	c.737delA,p Gln246Arg fsX10	NA	NED,29 months
Case 11		female	13	Surgical approach:NA Size: 16.9cm	NA	Her mother accepted hysterectomy for big leiomyoma at 32 years	Structure : Nested, tubulocystic, Cells : (grade 2) canalicular lumen	Negative	c.739 G>T,pGlu247 *	NA	NED,70 months
Case 12	2020/Lau et al (1case)	female	30	Surgical approach:NA Size:5.4cm	Incidental findings by MRI	Uterine leiomyomata RCC—family history leiomyomata—mother	Cells : Low-grade oncocytic	Negative	c.1189G> A, p. G397R	NA	NED,109 months
Case 13	2021/Wyvek enset al (1case)	female	30	Surgical approach:partial nephrectomy Size:2.0cm	Heavy menstrual cycles and abdominal/pelv ic pain	Uterine leiomyomata No family history	nested,tubular Cells : Low-grade oncocytic	Negative	p.P410L mutation	NA	NA
Case 14	2021/Hamza et al (1cases)	female	21	Surgical approach: radical nephrectomy Size:7.2cm	Screening due to family history dysmenorrhea,d yspareunia,and fatigue	Uterine leiomyomata Cutaneous Leiomyomata—mot her FH gene mutation—mother	Structure : Nested Cells : Low-grade oncocytic Focal mesenchyme : loose edema	Negative	Exon 6 deletion	NA	NED 6 months
Case 15	present case	male	48	Surgical approach: partial nephrectomy Size:2.1cm	Incidental findings by CT scan	None	Structure : Nested,tubular and microcysts Cells : Low-grade oncocytic(grade 2)	Negative	c.555+1G>C Intron 4 Splicing Mutation	adrenocortica l adenoma, 1.1cm (Clinical history: Presented with h i	NED 15 months
NED :no evidence of disease				NA, not available ;							

A growing body of data has led us to recognize and acknowledge the existence of low-grade tumors with FH-deficient RCC. FH-deficient low-grade morphology tumors are similar to high-grade morphology tumors in that they have a mixed tumor growth pattern, but current data on the former do not show a clear gender advantage, and their age of onset is relatively younger. In some cases, it was found incidentally by examination, and in a few cases, positive clinical signs did not reveal hematuria. Family history and evidence of HLRCC syndrome were reported in less than half of the cases; association with adrenocortical lesions was found in about 13.3% of the cases; 80% of the cases had a good prognosis at follow-up, and 20% had late onset of heterochronic high-grade or metastatic/death conditions. Based on the present published data, the histological term “low grade” is not synonymous with a good prognosis, as a few patients still experience poor outcomes. The microscopic morphology of this tumor is mostly described as low-grade eosinophilic, and the nuclear grade of the cells is mostly defined as ISUP/WHO 2016 grade 2, with occasional concomitant phenomena such as psammoma bodies, fatty components, and loose edema interstitium not excluded as suggestive for their diagnosis.

This rare low-grade form of FH-deficient RCC overlaps with succinate dehydrogenase (SDH)-deficient RCC and a variety of other low-grade eosinophilic renal tumors, further increasing the difficulty of differential diagnosis of this tumor. Wyvekens et al. listed the value of PAX8, CK7, CK20, CD117, TFE3, CathepsinK, SDH, FH and 2SC antibodies in the differentiation of various “eosinophilic” morphologically confusing renal tumors, such as FH-deficient RCC, SDH-deficient RCC, oncocytoma, eosinophilic solid cystic renal cell carcinoma (ESC-RCC), low-grade oncocytic tumor, (LOT), etc. (9). Hamza et al. summarized and outlined their experience in differentiating low-grade FH-deficient tumors from eosinophilic solid cystic renal cell carcinoma (ESC-RCC), low-grade oncocytic tumor (LOT), and eosinophilic vacuolated tumor (EVT) (5).

Pathological features play a key role in the diagnosis of FH-deficient RCC, but more experience is needed with low-grade morphologic tumors, and immunohistochemistry is a useful tool to differentiate this tumor from other renal tumors. Defective FH expression is a specific marker for the diagnosis of FH-deficient RCC; however, it has been found that FH-positive/complete immunohistochemical staining in a small number of cases does not exclude this diagnosis (9). The explanation for this phenomenon, given by some scholars through research, is that the FH staining in FH-deficient tumors is preserved in FH-deficient lesions with missense mutations, i.e., it is manifested as immunohistochemical FH positivity (4). Therefore, the combined use of 2-succinic acid cysteine (2SC) immunohistochemical assay is necessary in cases of FH-positive expression suspicious for this tumor (Loss of FH enzyme activity can cause intracellular fumarate production leading to high levels of 2SC production and subsequent positive 2SC IHC staining) (4, 5). The immunohistochemistry of 15 cases in this study of pure low-grade FH-deficient RCC showed negative expression of FH, and the existence of FH missense mutations/biallelic inactivation in this low-grade FH-deficient RCC needs to be further explored.

Detection of pathogenic FH mutations/biallelic inactivation is usually considered the gold standard for the diagnosis of FH deficiency. The updated WHO describes its pathogenesis as: Biallelic inactivation of FH. which encodes a Krebs cycle enzyme that converts fumarate to L-maleate, triggers oncometabolic fumarate accumulation, favouring aerobic glycolysis, decreasing oxidative phosphorylation, and perturbing several downstream pathways. In the current limited statistical data on low-grade FH-deficient RCC, the results of molecular testing of the FH gene reveal different mutation status in different cases (7). Certainly further FH molecular testing in patients with unsatisfactory immunohistochemical test results and suspicious clinical or pathological features is something to consider (4).

In conclusion, FH-deficient RCC is a rare entity with variable clinical and pathologic features, and a low-grade to high-grade morphological spectrum has been demonstrated. With the increasing empirical reaffirmation of purely low-grade FH-deficient RCC, it is easy to see that most patients with these tumors have a relatively good prognosis, but a few still have a poor prognosis and in some cases there is a family history and evidence of HLRCC syndrome. Additionally, it is important to note that biopsy of renal lesions in known or suspected HLRCC patients should be avoided. The spread of aggressive FH-deficient tumor cells during the procedure could occur, and implantation of metastatic lesions could occur as a direct result of the biopsy procedure. Therefore, low-threshold immunohistochemical screening for FH and/or 2SC is recommended when encountering difficult-to-classify RCCs, especially low-grade oncocytic renal tumors, in young patients.This is an important guideline for precise diagnosis and treatment, follow-up monitoring, and genetic counseling of low-grade FH-deficient RCCs.

## Data Availability

The original contributions presented in the study are included in the article/supplementary material. Further inquiries can be directed to the corresponding author/s.
